# An interactive simulator to deepen the understanding of Guyton’s venous return curve﻿

**DOI:** 10.1186/s12576-024-00912-9

**Published:** 2024-03-30

**Authors:** Noritaka Mamorita, Akihiro Takeuchi, Hirotoshi Kamata

**Affiliations:** 1 Department of Medical Informatics, Kitasato University School of Allied Health Sciences, 1-15-1 Kitasato, Minami-Ku, Sagamihara, Kanagawa 252-0373 Japan; 2 Department of Medical Informatics, Kitasato University School of Medicine, 1-15-1 Kitasato, Minami-Ku, Sagamihara, Kanagawa 252-0374 Japan; 3 Department of Hematology, Kitasato University School of Medicine, 1-15-1 Kitasato, Minami-Ku, Sagamihara, Kanagawa 252-0374 Japan

**Keywords:** Cardiovascular model, Venous return curve, Mean circulatory filling pressure, Interactive computer simulation, JavaScript, Web application

## Abstract

Mean circulatory filling pressure, venous return curve, and Guyton’s graphical analysis are basic concepts in cardiovascular physiology. However, some medical students may not know how to view and interpret or understand them adequately. To deepen students’ understanding of the graphical analysis, in place of having to perform live animal experiments, we developed an interactive cardiovascular simulator, as a self-learning tool, as a web application. The minimum closed-loop model consisted of a ventricle, an artery, resistance, and a vein, excluding venous resistance. The simulator consists of three modules: setting (parameters and simulation modes), calculation, and presentation. In the setting module, the user can interactively customize model parameters, compliances, resistance, *Emax* of the ventricular contractility, total blood volume, and unstressed volume. The hemodynamics are calculated in three phases: filling (late diastole), ejection (systole), and flow (early diastole). In response to the user’s settings, the simulator graphically presents the hemodynamics: the pressure–volume relations of the artery, vein, and ventricle, the venous return curves, and the stroke volume curves. The mean filling pressure is calculated at approximately 7 mmHg at the initial setting. The venous return curves, linear and concave, are dependent on the venous compliance. The hemodynamic equilibrium point is marked on the crossing point of venous return curve and the stroke volume curve. Users can interactively do discovery learning, and try and confirm their interests and get their questions answered about hemodynamic concepts by using the simulator.

## Background

Mean (circulatory) filling pressure (MFP), venous return curve (VRC), and Guyton’s graphical analysis are basic physiological and clinical concepts in cardiovascular hemodynamics [1–9]. MFP is defined as the mean vascular pressure that exists after a stop in cardiac output and redistribution of blood, so that all the pressures are the same throughout the system [1, 10]. In animal experiments, the heart was fibrillated, and mechanical occlusion has also been used. MFP was approximately 7 mmHg [1]. In theory, MFP is calculated by dividing the stressed blood volume by the compliance of the whole circulatory system including the compliance of the ventricle [8]. Most medical students conceptually understand MFP.

VRCs are brilliantly and simply displayed in many articles and medical textbooks [7, 9, 11, 12]. In these articles, VRCs were drawn as a straight line from the x-intercept (MFP, 0 flow) to the y-intercept (0 mmHg, high cardiac output). In the right-heart bypass preparation, Guyton’s original VRCs were obtained by procedures using a Starling collapsible tube and an external artificial pump. The procedures should be controlled stepwise raising the height of the tube and while controlling the pump rate to maintain a semi-collapsed state.

The use of the Starling resistor to vary the artificial pump rate has generated much confusion and little clarity. There continues to be ongoing debate on the interpretation of Guyton’s graphical analysis, ambiguity concerning the identification of the independent and dependent variables. What is a cause or an effect, i.e., “Pressure gradients from MFP to right atrial pressure was a consequence of flow, not determinants of flow.” [4, 5, 8, 13–17]. Subsequently, this controversy spread to the field of physiology education, i.e., “Guyton’s venous return curves should be/not be taught at medical schools” [8, 18].

In similar experiments excluding the Starling tube, when stepwise changes in the right atrial pressure were made only by the pump rate, similar VRCs could repeatedly be measured [13, 15]. Instead of animal experiments, medical students in Okayama University used a mechanical circuit model which consisted of elastic tubes and a pump without a Starling tube [19]. They obtained a pair of pressure-flow data points by increasing the pump rate manually in a stepwise fashion from zero to high. The VRCs were also drawn as straight lines from MFPs on x-intercepts under various conditions (Figs. 3–8 in Ref. [19]).

A simplified electrical model of the vascular system has been made based on six elements, three capacitances and three resistances without a Starling tube [2]. In the mathematical analysis, an algebraic formula for venous return was derived from the circuit analysis as a pressure gradient, e.g., an impedance to venous return. A closed-loop mathematical lumped model constructed from 16 linear equations was developed as an alternative to Guyton’s analysis relevant for heart failure [20]. The model was algebraically solved for dependent variables. Sunagawa et al. modeled the vascular system in those compliances, and the resistances were distributed in the systemic circulation. By a mathematical process, the equations derived were similar with those presented by Guyton [8]. Various computer simulators have been developed to teach physiology to deepen students’ understanding of MFP, VRC, and Guyton’s graphical analysis [8, 20–23]. VRCs were simply and clearly drawn as similar straight lines calculated by algebraic analyses.

Although mathematical analysis is straightforward and flawless, there might unfortunately be inadequate technical measuring of MFPs and VRCs. We not only want to show the results of VRCs, but the process of acquiring those results as well. We also want medical students to deeply consider these phenomena. We developed a pedagogical interactive simulator that accurately measures and clearly displays MFPs and VRCs with numerical iterations, not merely by solving algebraic differential equations.

## Methods

### The cardiovascular model and equations

**Fig. 1:**
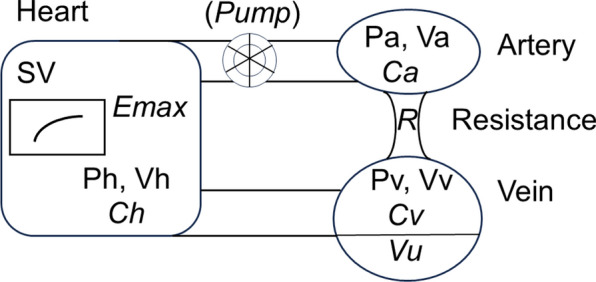
Lumped parameter, closed-loop model. The model consists of a ventricle, an artery, a resistance, and a vein. *Ca*, *Cv*, and *Ch* denote the arterial, venous, and ventricular compliances, respectively. *R* denotes the vascular resistance between the artery and the vein. P and V denote pressure and volume, respectively. SV denotes the stroke volume. The pump shown between the ventricle and artery is only attached in the VRC mode

The cardiovascular model is a lumped parameter, closed-loop minimalistic model based on physiological concepts. It consists of four elements: the ventricle, an artery, a vein, and a resistance (Fig. 1). The characteristics of the artery, vein, and ventricle are defined by pressure–volume (P–V) relations such as linear or non-linear. The resistance is put between the artery and the vein. Only during an experiment measuring VRC is the pump inserted between the heart (the ventricle) and the artery, and it is controlled, step by step, by the user clicking a button. Venous resistance and the Starling resistor are not defined. Total blood volume (*Vt*) consists of stressed volume (*Vs*) and unstressed volume (*Vu*). The *Vu* is only assumed to be in the vein for simplicity*.* Abbreviations are listed in Table 1.

**Table 1﻿:** Abbreviations of parameters and variables with their unit and default values

*Parameters﻿*		
*Vt*	Total bold volume	5100 mL^a^
*Vs*	Stressed blood volume	1200 mL^a^
*Vu*	Unstressed blood volume	3900 mL^a^
*R*	Resistance	1.5 mmHg/(ml/beat) ^a^
*Ca*	Arterial compliance	3.0 mL/mmHg for a linear function^a^
*Cv*	Venous compliance	150 mL/mmHg for a linear function^a^
*Emax*	Maximum elastance of ventricle	7 mmHg/mL^a^
*Ch*	Ventricular diastolic compliance	17 mL/mmHg
Variables		
Pa	Arterial pressure	mmHg
Va	Arterial volume	mL
Pv	Venous pressure	mmHg
Vv	Venous volume	mL
Ph	Ventricular pressure	mmHg
Vh	Ventricular volume	mL
EDP	End-diastolic pressure of ventricle	mmHg
EDV	End-diastolic volume of ventricle	mL
ESP	End-systolic pressure of ventricle	mmHg
ESV	End-systolic volume of ventricle	mL
SV	Stroke volume	mL/beat
F	Flow through the resistance	mL/beat
MFP	Mean filling pressure	
VRC	Venous return curve	
SVC	Stroke volume curve	

^a^These parameters can interactively be customized arbitrarily on the simulator

When P–V relations of the artery, the vein, and the ventricle were assumed as linear, the hemodynamic state of the circuit can be expressed with the algebraic equations:

1 $${\text{Vs}} = {\text{ Va}} + [{\text{Vv}} - Vu] + {\text{Vh,}}$$

2 $${\text{Pa}} = {\text{Va}}/Ca,$$

3 $${\text{Pv}} = [{\text{Vv}} - Vu]/{\text{Cv}},$$

4 $${\text{Ph}} = {\text{Vh}}/{{Ch,}}$$

5 $${\rm{F}} = [{\text{Pa}} - {\text{Pv}}]/R.$$

VRC is a relation of F and Pv and is algebraically derived as below. Substituting Va in Eq. (2), Vv in Eq. (3) and Vh in Eq. (4) into Eq. (1) yields:

6 $${Vs} = {\text{ Pa}} \times Ca + {\text{Pv}} \times Cv + {\text{Ph}} \times Ch.$$

Equation (5) is arranged for Pa:

7 $${\text{Pa}} = {\text{F}} \times R + {\text{Pv}}.$$

Substituting Pa in Eq. (7) into Eq. (6) yields:

8 $$\begin{aligned} {{Vs}} & = [{\text{F}} \times R + {\text{Pv}}] \times Ca + {\text{Pv}} \times Cv + {\text{Ph}} \times Ch \\ & = {\text{F}} \times R \times Ca + {\text{Pv}} \times Ca + {\text{Pv}} \times Cv + {\text{Ph}} \times Ch. \\ \end{aligned}$$

In an equilibrium state, Ph is the same as Pv in Eq. (8),

9 $$Vs = {\text{F}} \times R \times Ca + {\text{Pv}} \times [Ca + Cv + Ch].$$

Equation (9) is rearranged for Pv and F,

10 $${\text{F}} = [Vs - {\text{Pv}} \times (Ca + Cv + Ch)]/[R \times Ca].$$

When F = 0 in Eq. (10), Pv is equal to MFP,

11 $$0 = [Vs - {\text{Pv}} \times (Ca + Cv + Ch)]/[R \times Ca],$$

12 $${\text{Pv}} \times [Ca + Cv + Ch] = Vs,$$

13 $${\text{MFP}} = Vs / [Ca + Cv + Ch].$$

When Pv = 0 in Eq. (10), F is the y-intercept,

14 $${\text {F}}= Vs /[ R \times Ca].$$

Equations (10), (13), and (14) provide VRC, MFP, and y-intercept, respectively. The reciprocal of the slope of Eq. (10), [*R* × *Ca*]*/*[*Ca* + *Cv* + *Ch*] is the venous return resistance.

Based on algebraic formulas Eqs. (1)–(5), the simulator was developed to also handle non-linear P–V relations of the artery, vein, and ventricle. Non-linear functions are assumed as:

15 $${\text{Pa}} = A \times [exp (0.00{7} \times {\text{Va}}){-}{1}],$$

16 $${\text{Pv}} = B \times \left[ {exp \left( {0.00{1} \times \left[ {\text{Vv}} - Vu \right]} \right) - 1} \right],$$

17 $${\text{Ph}} = exp \left( {0.0{2} \times {\text{Vh}}} \right){-}{1,}$$

Coefficients of exponential functions, *A* and* B*, are initially set at 10 (mmHg/L) and 5 (mmHg/L), respectively. The *A* and *B* are customized within a determined range on the simulator. Program subfunctions calculate pressures from volumes and P–V curves. Inversely, subfunctions calculate the volumes from the pressures and P–V curves.

The ventricle works as a current source, not a pressure source. The current source can eject a certain volume against any resistive pressure of the artery in one beat. The ventricular function is based on Suga’s *Emax* model and a simple P–V loop [24]. The ventricle contracts at first as an isovolumic contraction at an end-diastolic volume (EDV). After the ventricular pressure exceeds a diastolic Pa, the ventricle contracts ideally as an isotonic contraction. When diastolic Pa is assumed as the end-systolic pressure (ESP), the end-systolic volume (ESV) is calculated by

$${\text{ESV}} = {\text{ESP}}/Emax.$$

Subsequently, stroke volume (SV) is naturally calculated as the difference between EDV and ESV.

### System overview

**Fig. 2:**
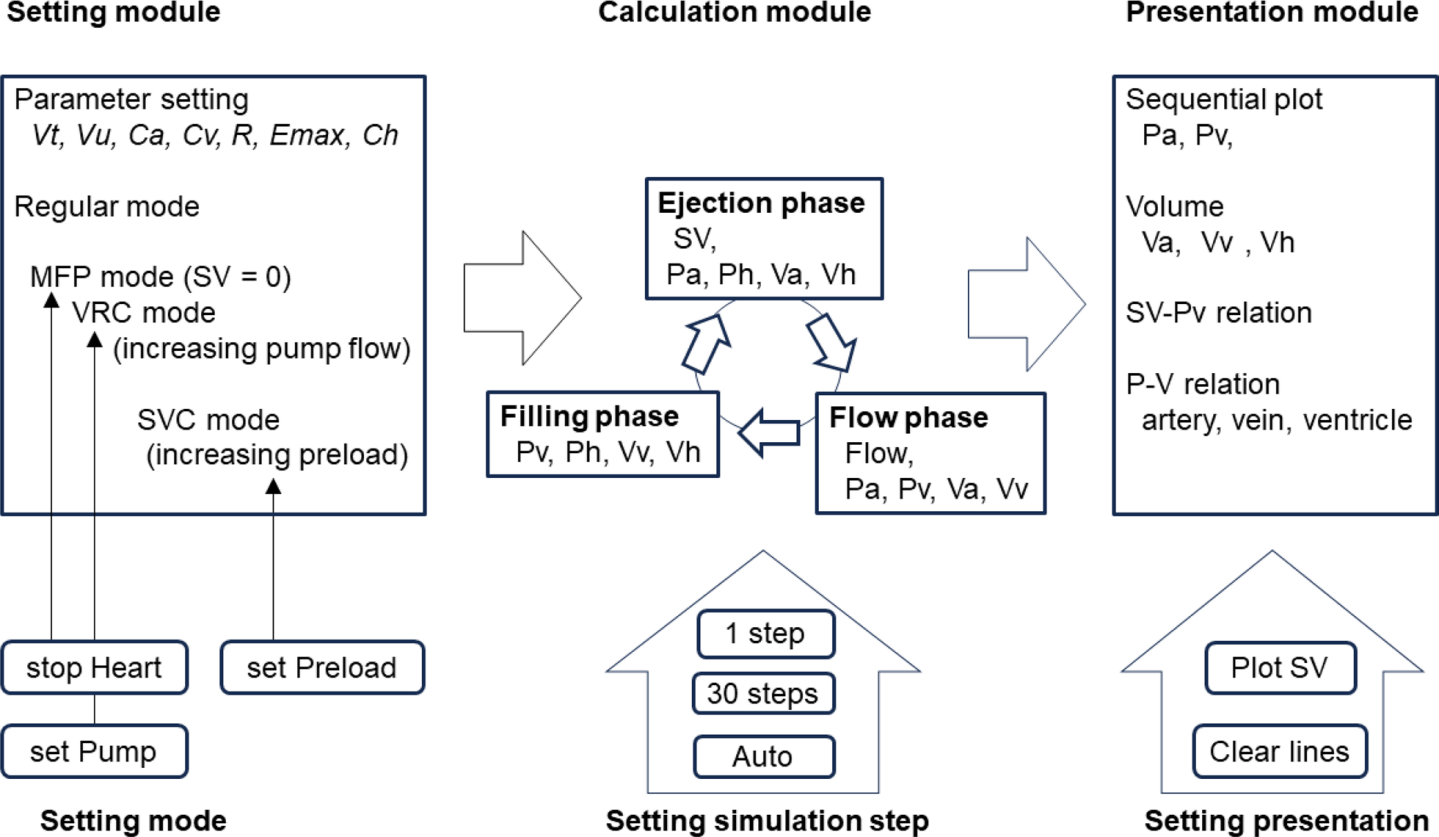
Simulator overview. The setting module is for setting values of parameters and simulation mode. The calculation module calculates hemodynamic states by three phases: the filling phase, the ejection phase, and the flow phase, in the simulation mode, regular mode, MFP mode, and the VRC mode. The calculation is achieved by the user’s click and timer events. The presentation module shows graphically Pa, Pv, Va, Vv, and Vh, and P–V relations of the artery, vein, and ventricle. Clicking buttons set the parameter value, simulation mode, simulation step, and the graph. One button, “Filling, Ejection, Flow”, processes a calculation step (phase) in the calculation module. The calculation is followed by the graphical presentation. The button, “Auto”, automatically causes timed events to occur without having to manually repeat the operation by repeatedly clicking the button

The simulator is designed to be used by medical students as a self-training tool. It is assumed that they are familiar with the basic concepts in cardiovascular physiology. The simulator consists of three modules: the setting module, the calculation module, and the presentation module (Fig. 2). These modules are controlled by the user’s commands shown at the lower part of the figure to set a mode, the simulation steps, and the presentation. In the setting module, the parameters of *Vt*, *Vu*, *Ca*, *Cv*, *R*, *Emax*, and *Ch* are interactively and arbitrarily modified by the user in a regular (default) mode. In the calculation module, hemodynamic states are calculated in three phases in one cycle: the filling phase (late diastole), the ejection phase (systole), and the flow phase (as early diastole). The calculation processes are described in the following section. The calculation phases are controlled by the user's commands, “1 step” and “30 steps”, and a timer clock (“Auto”). The presentation module updates graphs sequentially on the screen panel for each simulation phase.

The simulator has three modes in the regular (default) mode: the MFP mode, the VRC mode, and the stroke volume curve (SVC) mode. In the regular mode, the user can change the values of the parameters on the screen panel. Then new hemodynamic states are calculated and shown in the screen panel.

Other modes are set by the user’s commands shown at the lower area of Fig. 2. When the MFP mode is set by the button, “stop Heart”, SV is always set to zero flow because the ventricle is artificially arrested in animal experiments. The calculation and presentation are sequentially done the same as in the regular mode under a restricted condition, SV = 0 in the ejection phase. Blood in the artery, vein, and ventricle shifts due to the pressure gradients in the flow phase and the filling phase. The ventricle is expanded due to Pv but does not contract.

When the VRC mode is set by clicking the button, “set Pump”, an artificial pump is set between the ventricle and the artery. Although the ventricle is arrested, and therefore does not contract, it works as a conduit with a diastolic compliance. The pump flow is artificially increased in incremental steps of 10 mL by the user from 0 to 350 mL. The calculation and presentation are sequentially done the same as in the regular mode under a restricted condition, SV = pump flow in the ejection phase. The volume of the pump flow pulls blood from both the ventricle and the vein in the filling phase. In this simulator, the pump flow is set as a volume per beat (ml/beat) instead of a cardiac output (CO) (l/min). Points of Pv and pump flow are shown in the SV-Pv relation box.

When the SVC mode is set by clicking the button, “set Preload”, the ventricle is detached from the circuit model. SVs are solely calculated by increasing the preload under a constant *Emax* and afterload. When the preload Ph is sequentially set step by step from 0.5 mmHg to 9.5 mmHg, corresponding diastolic Vh measurements are calculated with an inverse subfunction of Eq. (17). As an afterload, the “current” Pa, which had been previously calculated, is used in the SVC mode. The SVC and the ventricular PV loop are plotted in the screen panel.

### Calculation module

Hemodynamic states are sequentially and periodically calculated in three phases for each cycle: the filling phase, the ejection phase, and the flow phase under the regular, MFP, and VRC modes.

#### Filling phase

Blood flows from the vein to the ventricle due to its pressure gradient. This blood shift, blood flow, is iteratively calculated for Pv and Ph to be in equilibrium.

When *“previous”* Ph < *“previous”* Pv*,* dV is set as 0.01 mL, or − 0.01 mL when “*previous”* Pv < *“previous”* Ph.

*“current”* Vv = *“previous”* Vv − dV.

*“current”* Vh = *“previous”* Vh + dV*.*

“C*urrent”* Pv and “current” Ph are calculated by subfunctions (Eqs. 3, 4, 16 and 17) with their P–V relations. These calculations are sequentially repeated until Pv and Ph are nearly the same.

*“Current”* values of Vh and Ph are used as *“previous”* values in the next ejection phase. And then *“Current”* values of Vv and Pv are used as *“previous”* values in the next flow phase.

#### Ejection phase

SV is derived by the ventricular function, preload Ph, and afterload Pa (diastolic Pa). The calculated SV is ejected from the ventricle to the artery. The SV is then added to the Va as in the common Windkessel model,

*“current”* Va = *“previous”* Va + SV.

*“current”* Vh = *“previous”* Vh − SV.

“C*urrent”* Pa is calculated by subfunctions (Eqs. 2 and 15) with the P–V relation of the artery. The increased Pa is a so-called systolic arterial pressure. *“Current”* Ph is calculated by subfunctions (Eqs. 4 and 17) with the P–V relation. *“Current”* values of Vh and Ph are used as *“previous”* values in the next filling phase. And then *“Current”* values of Va and Pa are used as *“previous”* values in the next flow phase.

#### Flow phase

A portion of arterial blood flows into the vein through the resistance by the pressure gradient between *“previous”* Pa and *“previous”* Pv. The flow volume is calculated with Eq. (5). New pressures and volumes are calculated as:

F = (*“previous”* Pa −* “previous”* Pv)/*R*.

*“current”* Va = *“previous”* Va − F.

*“current”* Vv = *“previous”* Vv + F.

“C*urrent”* Pa and Pv are calculated by subfunctions (Eqs. 2, 3, 15, and 16) with their *“current”* volumes. The decreased “*current*” Pa is a so-called diastolic pressure. *“Current”* values of Vv and Pv are used as *“previous”* values in the next filling phase. And then *“Current”* values of Va and Pa are used as *“previous”* values in the next ejection phase.

The hemodynamics in each simulation phase are sequentially calculated. The hemodynamic values reached the steady-state value from the initial value by iteration in the filling phase. The stable values are passed on as initial values of the next phase, filling to ejection, ejection to flow, and flow to filling. The calculations are repeated by the user’s command. When the user sets the MFP mode in any phase in the regular mode, the hemodynamic values at that time are passed on to the next phase. Although the SV is set to 0 mL in the ejection phase in the MFP mode, the calculations in other phases are repeated as they are in the regular mode. In the ejection phase of the VRC mode, SV is artificially set to the pump flow controlled by the user.

### Presentation module

The presentation module shows graphically hemodynamic states in every phase of the calculation module. Pa and Pv are continuously shown in each square box as a polygraph with numeric values. Va and Vv are also shown in vertical bars beside the Pa and Pv boxes in numeric values. SV and Pv points are shown in the SV-Pv box. The ventricle is shown as an animated circle, expanding and contracting with values of Vh and Ph.

For compliance, easy to understand at a glance, three P–V curves are shown as straight lines or exponential curves in three P–V relation boxes. Current points of pressure and volume are also marked with various colored circles on those lines and curves. In the ventricular P–V relation box, current points of (EDP, EDV) are shown. In the MFC and VRC modes, to express the ventricular filling, the diastolic curve is magnified 20-fold and drawn in blue lines because the diastolic characteristics of the ventricle are drawn in relatively flat lines near the x-axis. In the SVC mode, the PV loops of the ventricle are drawn.

In the MFP mode, the ventricular command stops contracting action but does expand due to Pv. Moreover, in the VRC mode, the animation pump is set between the ventricle and the artery. Points of (Pv, SV) are shown in the SV-Pv box. The VRC and SVC are plotted in the SV–Pv box. The crossing point of the VRC and the SVC indicates the equilibrium point of the hemodynamics.

### Application coding and URL

This simulation system was developed as a web application. The application is integrated into an HTML file including SVG (scalable vector graphics) code and JavaScript codes (about 3000 lines, 100 kB in total) and can be accessed at “https://www.zmondai.com/p/vrc.html”.

## Results

### The user interface of the simulator

**Fig. 3:**
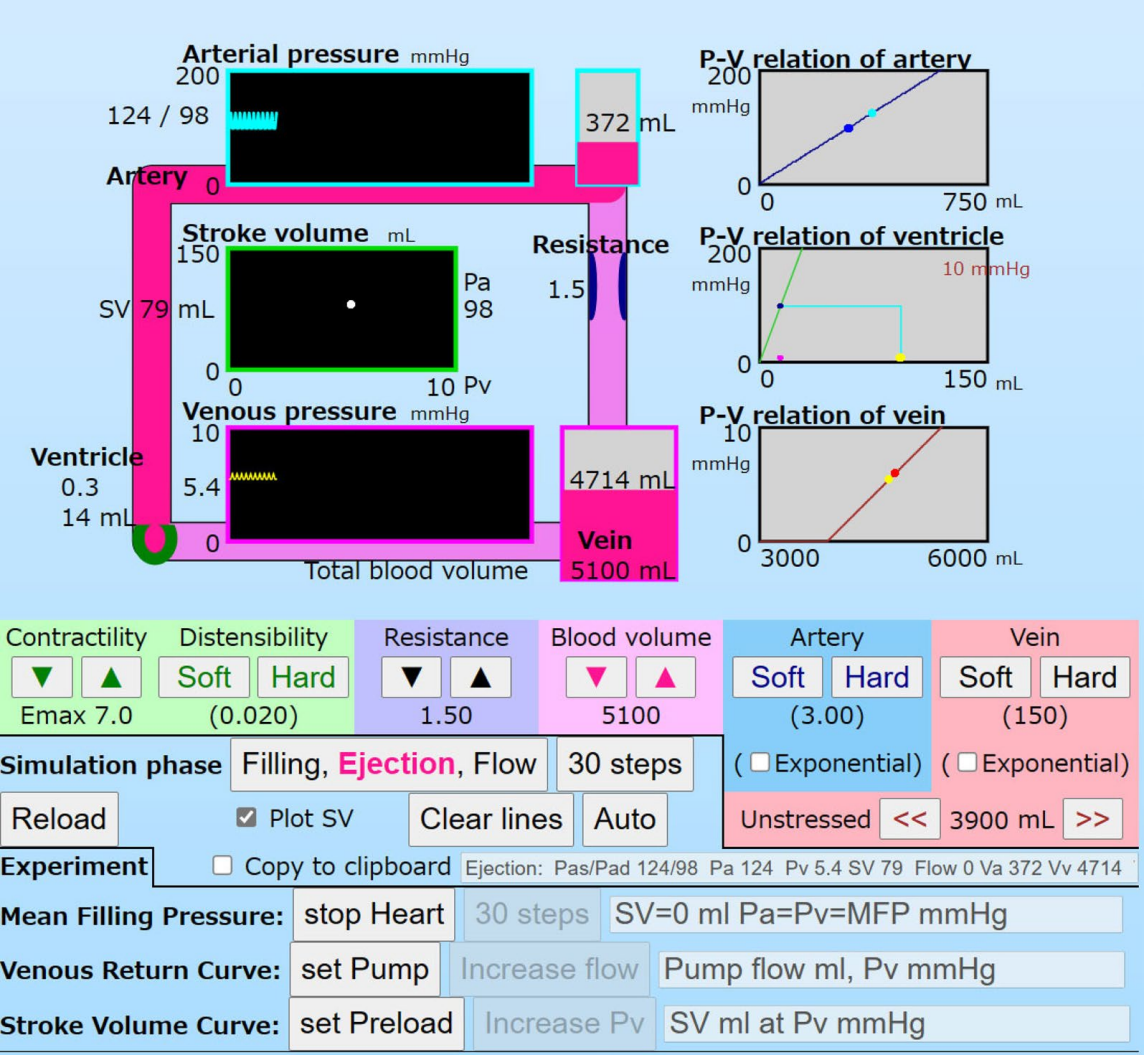
A screen shot of the simulator in the regular mode. The circuit model is shown with pink pipes at the upper left side. The heart is indicated as a green circle (horseshoe shape) at the lower left corner of the circuit. The SV-Pv box is set at the center of the circuit. On the right, there are P–V relation boxes that show each compliance and current point (volume, pressure) by calculation. In the middle, there are control buttons for ventricular contractility and distensibility, resistance, blood volume, arterial and venous compliances, and unstressed volume. In the lower area, there are buttons for the simulation mode and speed. The button, “Filling, Ejection, Flow” is for a calculation step. The red caption of the button shows, step by step, the phase of the three phases: “30 steps” is for 30 calculation steps, “Auto” is for staring the timer clock, and “Clear lines” is for clearing points and lines complicated in plot areas. “Reload” is for loading the simulator application to restart the stimulator. A checkbox, “Plot SV” is for plotting SV in the SV-Pv box. “Copy to clipboard” is for recording simulation processes for convenience. The buttons, “stop Heart”, “set Pump”, and “set Preload” are for setting experiment conditions: the MFP mode, the VRC mode, and the SVC mode. The simulation for each mode is done the same as in the regular mode

Figure 3 is a screen shot of the simulator. There is a circuit model on the left, P–V relation boxes on the right, and setting buttons for parameters and simulation modes in the lower area. In the circulation model, the ventricle is set in the left lower corner as a green circle (horseshoe shape) that expands and contracts in the filling and ejection phases. The artery and the vein are filled with blood indicated in pink. Blood shifts are animated by movement of pink (blood) on the circuit. The resistance is vertically set between the artery and the vein. The blood volumes in the artery and vein are drawn on each vertical bar adjacent to the panels for Pa and Pv. Pressures in the artery and vein are continuously shown in cyan and yellow, respectively. SVs are shown in the middle box with a green frame.

On three gray panels in the right area, P–V relations, linear and exponential functions, are drawn with current points at every simulation phase. The arterial pressure in the systole and diastole are shown in cyan and blue, respectively. The maximum and minimum venous pressures are shown in orange and yellow, respectively.

In the lower area, there are control buttons to increase or decrease *Emax*, *Ch*, *R*, *Vt*, *Vu*, *Ca*, and *Cv*. There are also control buttons to set the simulation steps: “1 step” (“Filling, Ejection, Flow”), “30 steps” (10 cycles), and “Auto” (continuous simulation), to clear lines and markers drawn in certain boxes (“Clear lines”), to reload the application (“Reload”).

There are three buttons for MFP mode, VRC mode and SVC mode. A button, “stop Heart” is for MFP mode to cause the ventricular arrest for measuring the MFP. A button, “set Pump” is for the VRC mode to cause the ventricular arrest and to set a pump for measuring VRC. By clicking that button, the VRC mode is set and the button, “increase pump flow” is shown. User can increase the pump flow incrementally and watch the simulated hemodynamics, Pa, Pv, Pump flow (SV), Va, Vv, and Vh. A button, “set Preload” is for the SVC mode to extract the ventricle from the circuit, and to calculate the ventricular function solely. The preload (Pv) is controlled from 0.5 to 9.5 mmHg. The values of the *Emax* and afterload (diastolic Pa) in the previous regular mode are used.

### Simulations

The hemodynamics were calculated in three phases of a cardiac cycle. Under the initial setting in the regular mode, a stable hemodynamic state is shown in the first section of Table 2. In the ejection phase, SV 79 ml was ejected from the ventricle into the artery. Then Va was increased from 293 to 372 mL, and Pa increased from 98 to 124 mmHg. In the flow phase, 79 mL flowed from the artery to the vein. Vv increased from 4714 to 4793 mL. Pv increased from 5.4 to 6.0 mmHg. In the filling phase, Vh increased from 14 to 93 mL due to the pressure difference between Pv and Ph. Then Ph increased from 0.3 to 5.4 mmHg and Pv decreased. Pv, Va, Vv, Ph, and Vh pulsated as Pa in the simulation phases. These phenomena were animated with the ventricular beating, blood shifts, and fluctuations of volume bars.

**Table 2﻿:** Volumes and pressures at the steady state of simulation phases and modes

Condition Mode and phase	Pa mmHg	Pv mmHg	Ph mmHg	SV mL^a^	Flow mL^a^	Va mL	Vv mL	Vh mL
*Emax* 7.0								
Regular mode								
Filling	98	5.4	5.4	0	0	293	4714	93
Ejection	124	5.4	0.3	79	0	372	4714	14
Flow	98	6.0	0.3	0	79	293	4793	14
MFP mode (SV was set 0 mL in the ejection phase)								
Filling, ejection, flow	7.2	7.2	7.2	0	0	21	4974	105
VRC mode (for the pump flow was set to 350 mL^a^)								
Filling	408	0.0	0	0	0	1225	3875	0
Pump flow	525	0.0	0	350	0	1575	3525	0
Flow	408	0.0	0	0	350	1225	3875	0
*Emax* 2.0								
Regular mode								
Filling	75	5.9	5.9	0	0	224	4780	96
Ejection	94	5.9	1.1	59	0	283	4780	37
Flow	75	6.3	1.1	0	59	224	4839	37
MFP mode								
Filling, ejection, flow	7.2	7.2	7.2	0	0	21	4974	105

^a^Per beat

**Fig. 4:**
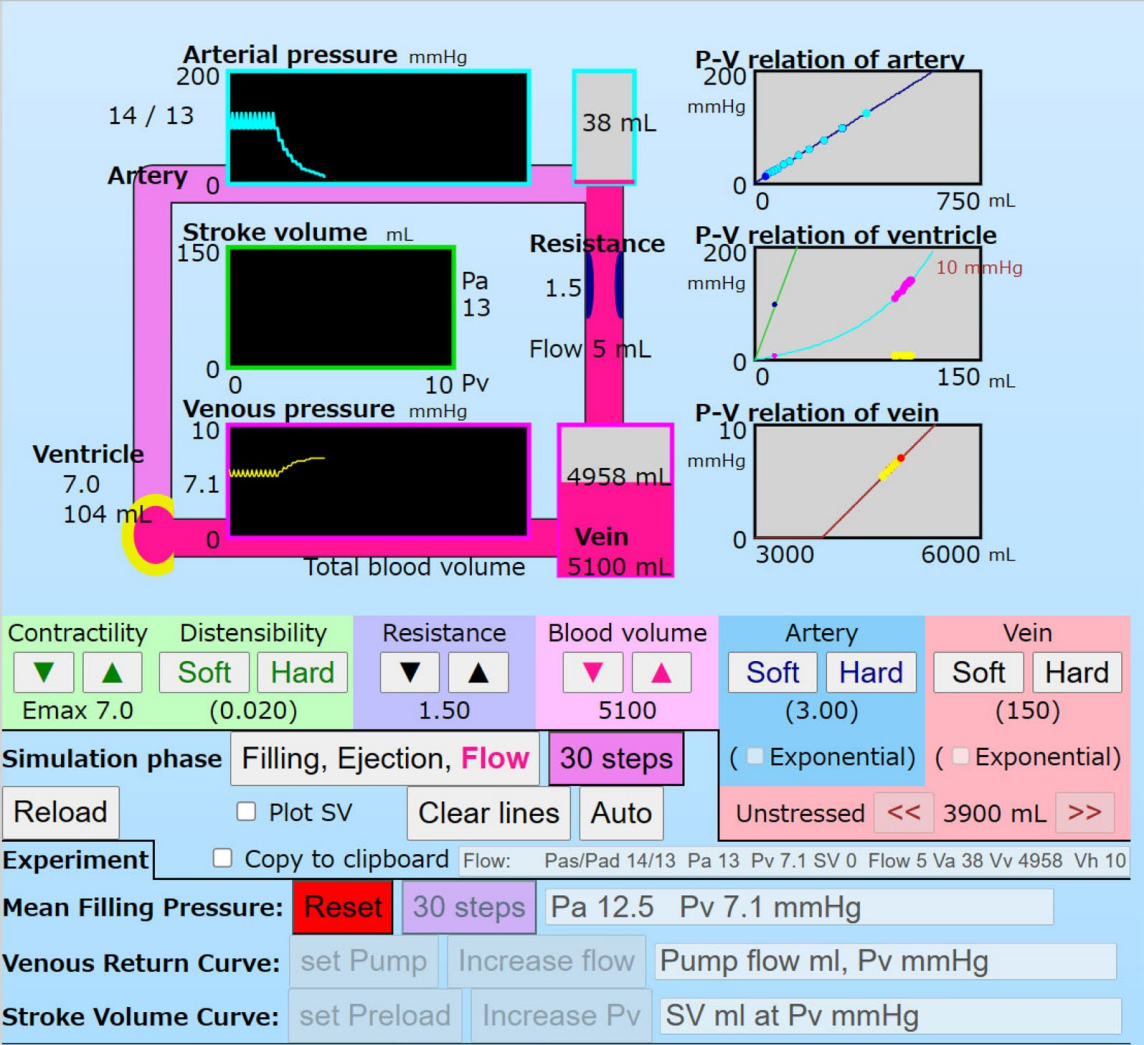
A screen shot of the MFP mode. Decreasing Pa and increasing Pv are shown in cyan and yellow. Blood in the artery shifts to the vein in pink. The ventricle, green circle (horseshoe shape) of the regular mode is colored yellow, which means arrest: no ejection but expanding due to Pv. The P–V relation box of the ventricle shows a diastolic curve magnified 20-fold to express the current point (Vh, Ph). Clicking the button, “Filling, Ejection, Flow” processes, step by step, a phase the same as in the regular mode. Clicking the button, “30 steps” processes 10 cycles of the calculation. At the lower area, a caption of the button, “stop Heart” was changed to “Reset” with red indicating going back to the regular mode

**Fig. 5﻿:**
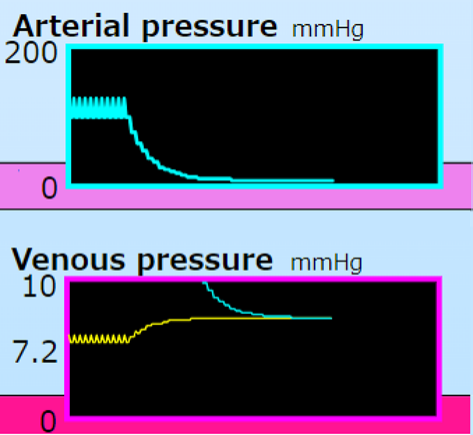
A part of the simulator panel in a case of the MFP mode. Pa is shown as a cyan line in the Pa and Pv boxes. Pv is shown as a yellow line in the Pv box

The MFP mode is set by clicking the button, “stop Heart” on the right, “Mean Filling Pressure” (Fig. 4). The button was changed to, “Reset” with red indicating going back to the regular mode. The SV was set as 0 at every ejection phase in the calculation. Pa decreased, step by step, for each cycle from 124/98 to 7 mmHg (Fig. 5). Inversely, Pv increased from 6.0/5.4 to 7.2 mmHg. Pa, Pv, and Ph were finally equivalent without pulsation. The MFP was 7.2 mmHg in this case (Table 2).

**Fig. 6:**
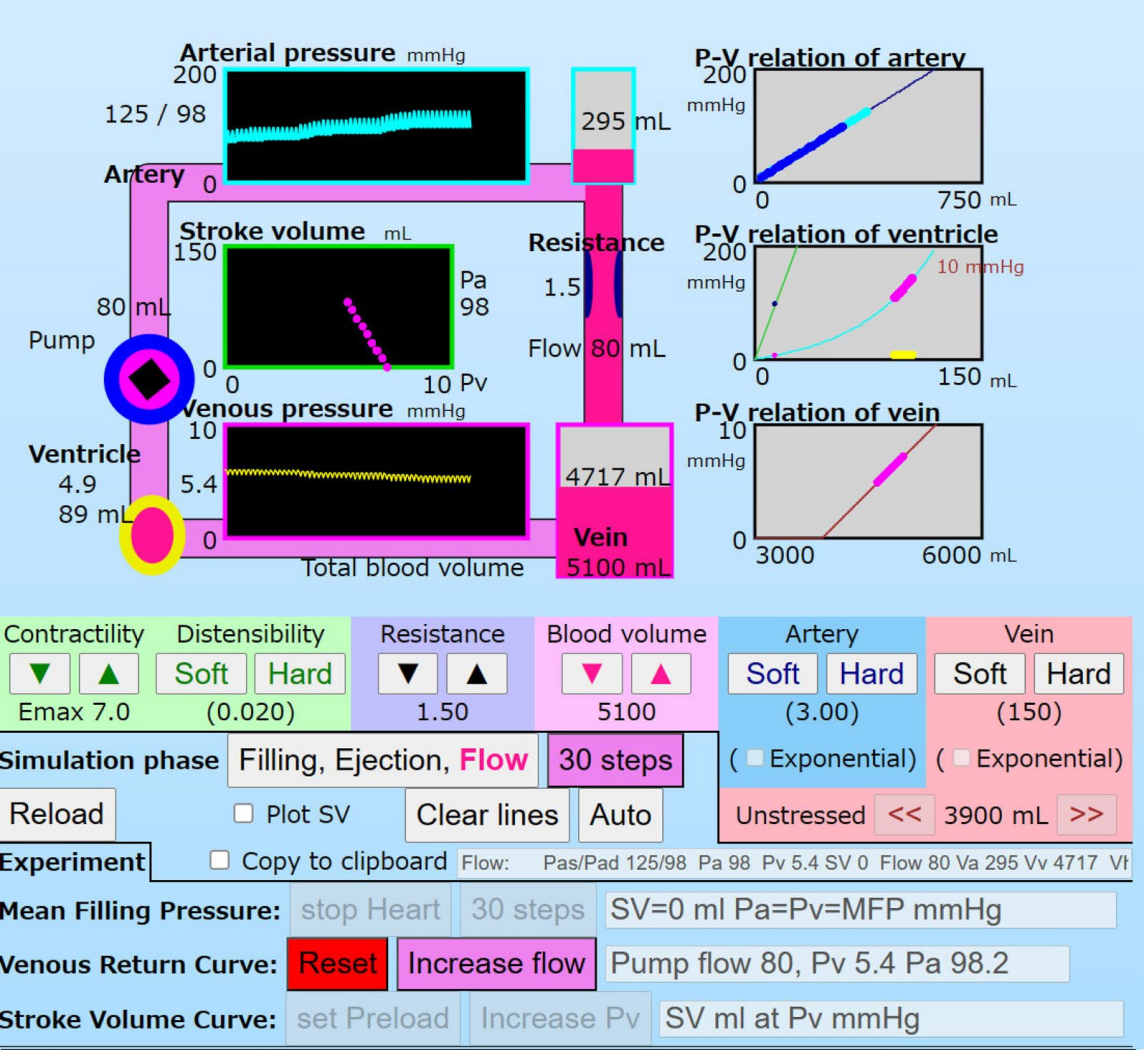
A screen shot of the VRC mode. The button “increase flow” is prominently displayed in the lower area. The pump is animated (the circled diamond with a blue circle) above the heart. When the pump flow is increased, step by step, to 80 mL in this case, Pa increased, and Pv decreased. The ventricle becomes smaller due to the decreasing Pv. The P–V relation box of the ventricle shows a diastolic curve to express the current point (Vh, Ph), the same as that in the MFP mode

The VRC mode is set by clicking the button, “set Pump” on the right, “Venous Return Curve” in the regular mode (Fig. 6). When the pump flow is increased step by step from 0 to 350 mL, Pa increased and Pv decreased. Points (Pv, pump flow [SV]) were sequentially plotted in the SV-Pv box. A case of the pump flow at 80 mL/beat is shown. Pv decreased from 7.2 (MFP) to 5.4 mmHg. Pa increased from 7 to 125/98 mmHg. Vv decreased from 4972 to 4717 mL. Va increased from 22 to 295 mL. The P–V relation box of the ventricle shows a diastolic curve magnified 20-fold to express the current point (Vh, Ph) as same as the MFP mode. The PV loop of the heart is not shown due to the ventricular arrest.

**Fig. 7:**
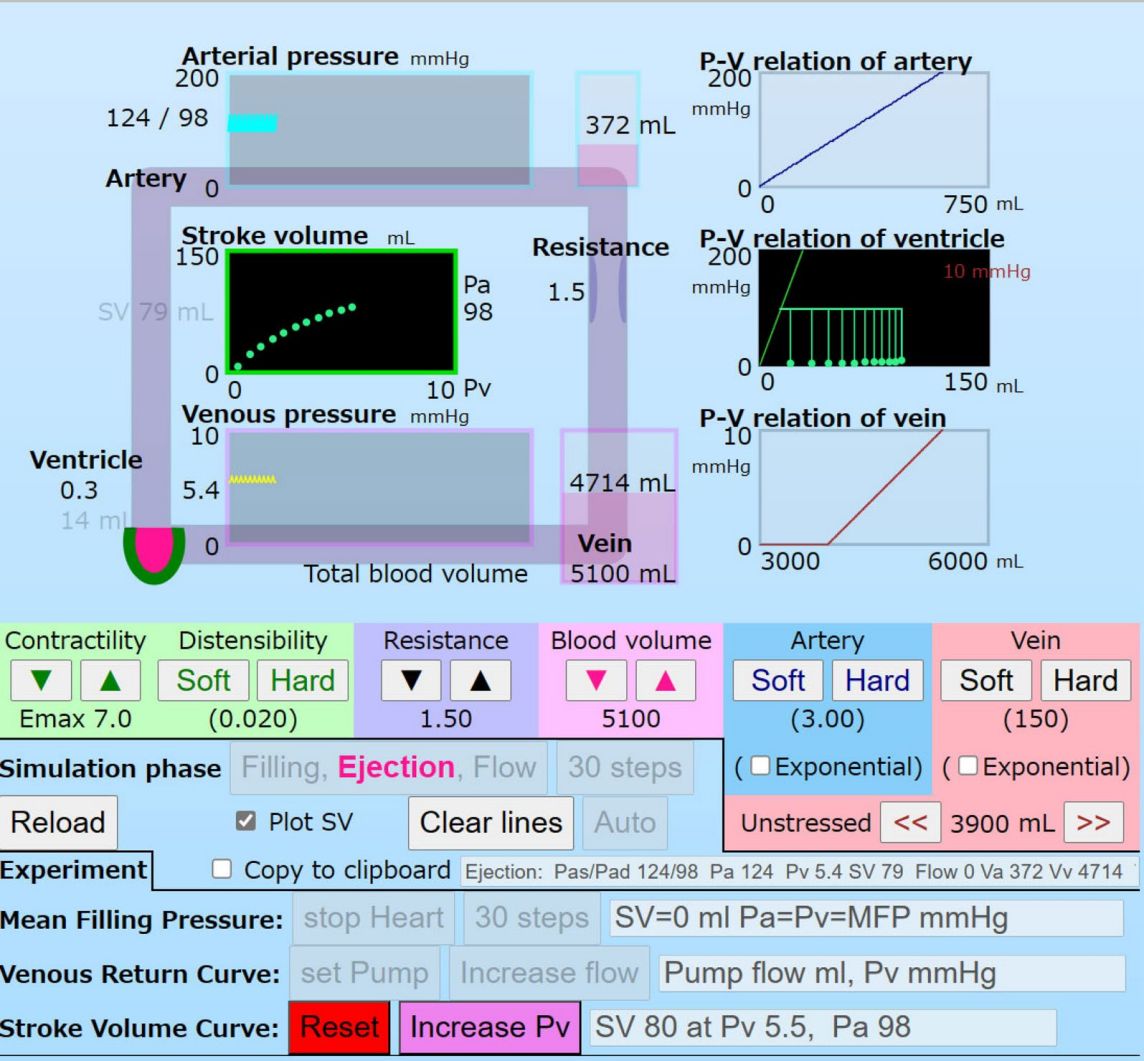
A screen shot of the SVC mode. The button “Increase Pv” is prominently displayed at the lower area. The circuit in a light pink and the P–V relation box of the artery and vein are expressed in a light color in the SVC mode. The ventricle with a green circle (horseshoe shape), SV–PV box, and P–V relation box are prominently displayed. When the preload of the ventricle, Pv, is increased step by step, SVC and PV loops are drawn in the boxes

The SVC mode is set by clicking the button, “set Preload” on the right, “Stroke Volume Curve” in the regular mode (Fig. 7). SVC and PV loops of the ventricle are shown in Fig. 7. SVs were calculated by increasing the preload, Ph, under a constant contractility *Emax* and the afterload Pa 98 mmHg. The Pa was previously calculated in the regular mode. SVC was a convex curve and directly affected by the ventricular diastolic compliance, P–V relation.

**Fig. 8﻿:**
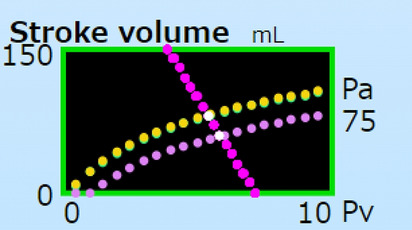
Effects of *Emax* on SVC and VRC. The SVC and VRC are drawn overlapping in the SV–Pv box at the SVC mode and VRC mode, respectively. Contractility *Emax* 7.0 and 2.0 affected SVCs, as shown in yellow and purple, respectively. The VRC was not affected. The Pa value of 75 presented is for *Emax* 2.0. Although the Pa value of 98 is for *Emax* 7.0, it was overwritten and, therefore, is not shown

To show the effects of the contractility *Emax* on VRC, as a simulation of heart failure, *Emax* is abruptly reduced from 7.0 to 2.0 mmHg/mL (Fig. 8). The hemodynamics are summarized in the second section of Table 2. SV decreased from 79 to 59 mL. Pa decreased from 124/98 to 94/75 mmHg. Inversely, Pv increased from 6.0/5.4 to 6.3/5.9 mmHg. Although two VRCs were each drawn in the simulator, those two VRCs were completely overlapping in the figure. This means that the *Emax* does not affect the VRC. Crossing white points of the SVCs and the VRC showed equilibrium points of two hemodynamic states. The SV-Pv box are used to plot current points (Pv, SV). Those points are not deleted until the user clicks the button, “Clear lines”. Then the user can simultaneously watch the VRC and SVC, crossing any other curves at the hemodynamic steady-state point.

**Fig. 9:**
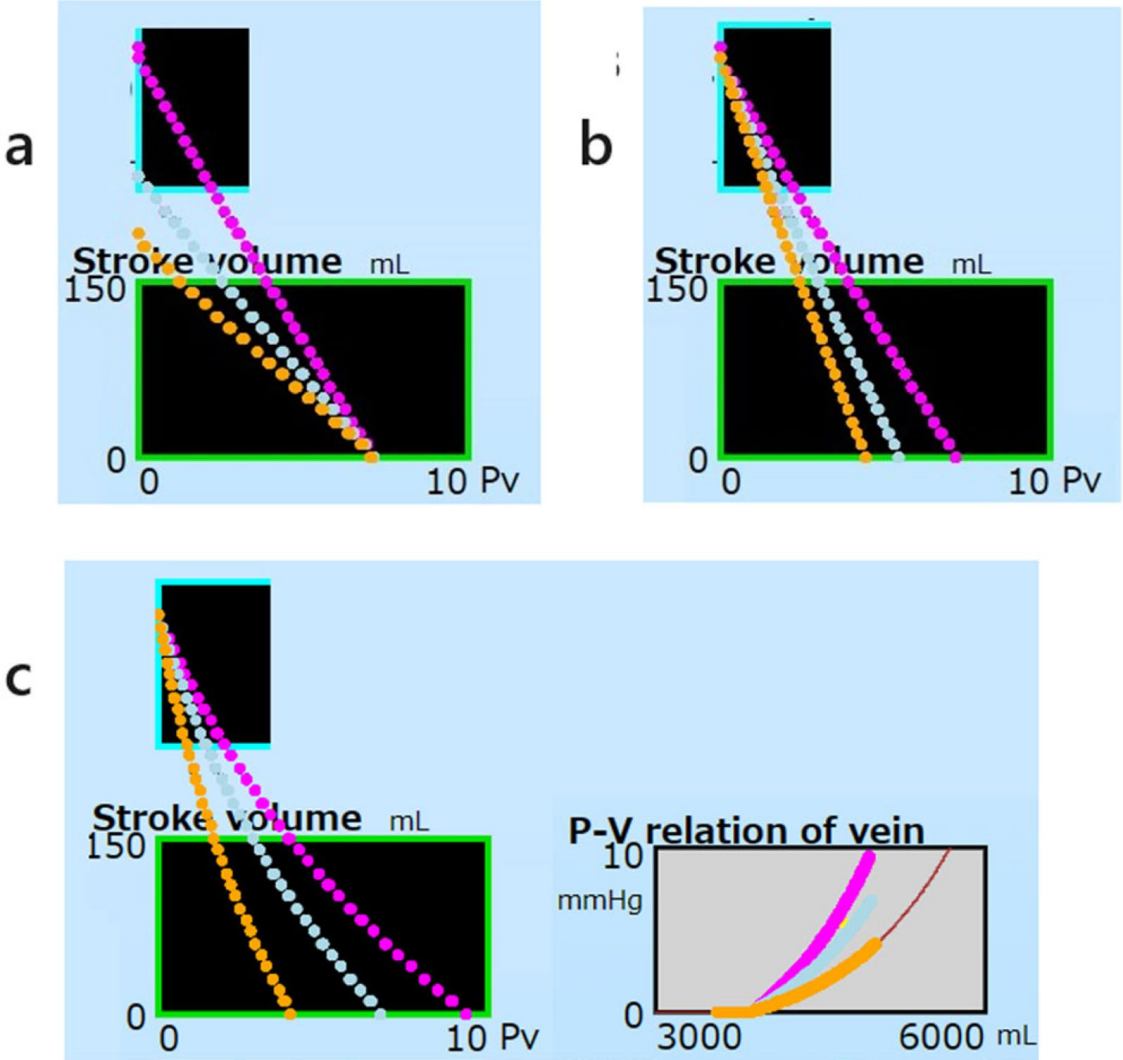
Effects on VRC of *R*, *Cv* for linear P–V relations, and the coefficient *B* of Eq. 16 for non-linear P–V relations. Magenta, light blue, and orange indicate various conditions. a *R* was set at 1.5, 2.0, and 2.5 (mmHg/[mL/beat]). **b** *Cv* in Eq. 3 was set at 150, 200, and 250 (mL/mmHg). **c** The coefficient *B* of Eq. 16 was set at 5.0, 3.5, and 2.0. Three compliance curves in the P–V relation panel correspond to the colored VRCs

Effects of the parameter *R*, linear and non-linear *Cv* on VRCs are shown in magenta, light blue, and orange, respectively, in Fig. 9. Values of MFP, Va, and Vv in the MFP mode, as well as the volumes and Pa at the y-intercept, for each parameter set are listed in Table 3. Variations of the resistance from 1.5 to 2.5 (mmHg/[mL/beat]), did not affect the MFP at 7.2 mmHg. The VRCs were linear with various slopes (Fig. 9a). The y-intercepts varied from 350 to 190 mL/beat. When the y-intercept was reached by increasing pump flow, calculated arterial pressures became unrealistically high. When venous compliance was softened from 150 to 250 (mL/mmHg), the MFPs decreased from 7.2 to 4.4 mmHg. Those three VRCs were linear (Fig. 9b). When the venous compliance was softened by decreasing the coefficient of Eq. 12 from 5.0 to 2.0, the MFPs decreased from 9.4 to 4.0 mmHg. The y-intercepts were the same at 350 mL/beat. The VRCs and P–V relations of the vein are shown as concave curves (Fig. 9c). The P–V relation of the vein is reflected on the shape of the VRC. Softening of the vein, even in linear and non-linear modes, lowered the MFP.

**Table 3﻿:** MFP and y-intercept of VRC in various conditions

	MFP mmHg	Va mL	Vv mL	Vh mL	Y-intercept- mL	Va mL	Vv mL	Vh mL	Pas/Pad^b^ mmHg
Setting condition									
*R*									
1.5^a^	7.2	21	4974	105	350	1225	3875	0	525/408
2.0	7.2	21	4974	105	240	1199	3901	0	480/400
2.5	7.2	21	4974	105	190	1234	3866	0	475/411
*Cv* in Eq. 3 for a linear P–V relation to the vein									
150^a^	7.2	21	4974	105	350	1225	3875	0	525/408
200	5.5	16	4990	94	350	1225	3875	0	525/408
250	4.4	13	5002	85	350	1225	3875	0	525/408
*Cv*, coefficient B of Eq. 16 for a non-linear P–V relation to the vein									
5.0^a^	9.4	28	4955	117	350	1225	3875	0	525/408
3.5	6.8	20	4977	103	350	1225	3875	0	525/408
2.0	4.0	12	5007	81.0	350	1225	3875	0	525/408

^a^Initial setting value

^b^Pas and Pad are Pa in systole and diastole, respectively

## Discussion

This simulator was developed for medical students to theoretically consider various cardiovascular states. The systemic circulation of the simulator consists of *Ca*, *R*, and *Cv*, without venous resistance. For systemic circulation, Guyton’s original model (1955) consisted of six components: three capacitances and three resistances. Guyton’s simplified model (1959) consisted of four elements: arterial capacitance and resistance, and venous capacitances and resistance [5, 25]. The formulas for MFP and venous return (VR) were algebraically derived as:

18 $${\text{MFP}} = [{\text{Va}} + ({\text{Vv}} - Vu)]/[Ca + Cv],$$

19 $${\text{VR}} = \left[ {\text{MFP}} - {\text{Pra}} \right] / \left[ Rv + Ra \times \left( Ca / \left[ Ca + Cv \right] \right) \right],$$

where Pra is the right atrial pressure, and *Ra* and *Rv* are the arterial and venous resistances, respectively. Although MFP was not an exist pressure source on the circuit [8], the equation of MFP was derived by dividing the total stressed volumes by the total compliances.

Equations (13) and (18) were conceptually similar. In Eqs. (10) and (19), Pv and F, and Pra and VR, were rewritten, respectively.

20 $${\text{F}} = \left[ ({\text{Va}} + \right[{\text{Vv}} - Vu \left] + {\text{Vh}}){-}{\text{Pv}} \times \left( Ca + Cv + Ch \right)]/[R \times Ca \right],$$

21 $${\text{VR}} = \left[ {\text{Va}} + \left( {\text{Vv}} - Vu \right) - {\text{Pra}} \times \left( Ca + Cv \right) \right] / \left[ Rv \times \left( Ca + Cv \right) + \left( Ra \times Ca \right) \right].$$

Equations (20) and (21) were conceptually similar, other than the Rv term.

Non-linear elements increase model complexity, in which case, numeric methods become necessary to solve the model equations. The present simulator treated not only linear parameters but also optionally non-linear functions for *Ca*, *Cv*, and *Ch*. When an exponential compliance is set on the vein, its VRC is concaved from a point on the MFP (Fig. 9). In Guyton’s result (Fig. 2 in Ref. [3]), the lower part of the VRC, near the MFP, appeared somewhat as a concave curve rather than a linear line. Noteworthy, a linear VRC meant that all the venous vessels would present as though they had linear characteristics.

Most models were simple representations of the actual physiological complex system [5]. Although our minimal model consists of four elements, the simulator showed experimental processes of the SVC and VRC, step by step, simply by the user’s click. A graph of the SVC and VRC reminds the reader that the cardiovascular system model contains both a heart and the circulation system but does not tell the whole story of how the system behaves [14]. Regarding the results, the y-intercept, Pv = 0, naturally means that all of the stressed volume in the vein should be shifted to the artery. It naturally causes Pa to be significantly increased. However, to our knowledge, Guyton did not report the changes in arterial pressure that occur with changes in cardiac output in his experiments [14].

A Starling resistor is not necessary to measure the VRC. Similar VRCs without a Starling resistor have been reported [13, 15]. VRCs were drawn as straight lines when a mechanical circuit model consisted of elastic tubes and a pump without a Starling resistor [19]. Therefore, the model of the simulator does not include a Starling resistor.

Although Guyton defined the “venous resistance” between the venous compliance and the heart, the effect of the venous resistance on the VRC is not easily, nor explicitly, visualized in his diagram [26]. The venous resistance could not realistically be imaged. It is not clear where the upstream point of the venous resistance to the atrium is. Although models and simulators may generally have limitations, this model excludes the venous resistance to grasp the understanding of the basic theory of hemodynamics more easily.

Even though the simulator showed usual VRCs, the simulator showed unexpectedly high values of the y-intercept of VRCs accompanying high values of Pa. The y-intercept of the VRC based on Guyton’s simplified model is algebraically derived from Eq. 21,

$${\text{Y - intercept}} = [ {\text{Va}} + ( {\text{Vv}} - Vu ) ] / \left[ Rv \times \left( Ca + Cv \right) + \left( Ra \times Ca \right) \right].$$

This equation emphasizes that the y-intercept increases naturally when *Rv* is zero. Then the high y-intercept is considered as a result by excluding any venous resistance from the circuit model. Although the model may be revised to include the venous resistance for a more realistic effect, and ought to show the effects of venous resistance on the y-intercept, those concepts warrant future study.

In congestive heart failure, cardiac contractility is compromised. As a result, mean arterial pressure decreases and central venous pressure increases [5]. This phenomenon was well simulated by decreasing *Emax* in the present simulator (Table 2, Fig. 6). However, there was a phenomenon that this model could not simulate. Usually, when the afterload increases, the venous pressure will likely increase as well because the cardiac output will decrease if the contractility is not changed. In the simulator, when *R* was increased, blood in *Ca* was dammed up by the resistance, Va and Pa increased, and conversely Vv and Pv decreased due to a decrease in inflow from the artery to the vein. Subsequently, a more complex model became necessary for this phenomenon.

Because most physiological systems are complex, simplification helps to facilitate the interpretations of the predictions generated by a model [5]. An approximate model is valuable for two reasons: it provides a cogent tool with which we can more easily think about a complex system, and it generates ideas that can be tested [5]. The simulator may be useful to understand hemodynamics and effects by the changes in parameters on hemodynamic variables. “The cardiovascular system” consists of the heart and the vessels. The user may recognize that SVC and VRC characterizes the heart and the vascular system, respectively. The hemodynamic steady-state in the closed-loop model is understood as the intersection of SVC and VRC shown in the simulator. Although the effectiveness of the simulator was personally developed for medical students who have scant knowledge about “venous return” and “cardiac output”, it is difficult, and not realistic, to objectively evaluate the simulator in the medical school environment. Although the effectiveness of new educational tools is generally expected to be able to be proven at anytime, a long-term follow-up of the simulator's effectiveness was not done, but rather the focus of the present study was on a means of assisting students’ active learning. The simulator application can now be accessed online, at no cost, for medical students and new cardiovascular clinicians as well. Guyton advocated discovery learning [14]. Users can interactively try to get answers and confirm their interests on the simulator. For medical students, the process of developing a reasonable answer to each of their questions is often more important than the answers themselves.

## Conclusions

An interactive cardiovascular simulator was developed for medical students to get a better grasp of “Guyton’s graphical analysis”. Users can interactively vary the model parameters, compliances, resistance, *Emax*, total blood volumes, and *Vu* to construct their own self-tooled learning experiments. Hemodynamics, MFP, VRC, SVC, and equilibrium points are simulated and graphically presented. This simulator may be helpful for students to deepen their understanding of the concepts and principles of cardiovascular hemodynamics.

### Availability of the simulator

This simulator is publicly available as a web application at https://zmondai.com/p/vrc.html.

## Data Availability

Not applicable.
